# An image‐based model of brain volume biomarker changes in Huntington's disease

**DOI:** 10.1002/acn3.558

**Published:** 2018-04-02

**Authors:** Peter A. Wijeratne, Alexandra L. Young, Neil P. Oxtoby, Razvan V. Marinescu, Nicholas C. Firth, Eileanoir B. Johnson, Amrita Mohan, Cristina Sampaio, Rachael I. Scahill, Sarah J. Tabrizi, Daniel C. Alexander

**Affiliations:** ^1^ Department of Computer Science Centre for Medical Image Computing University College London Gower Street London WC1E 6BT United Kingdom; ^2^ Huntington's Disease Research Centre University College London 2nd Floor Russell Square House, 10‐12 Russell Square London WC1B 5EH United Kingdom; ^3^ CHDI Management/CHDI Foundation 350 7th Avenue New York New York

## Abstract

**Objective:**

Determining the sequence in which Huntington's disease biomarkers become abnormal can provide important insights into the disease progression and a quantitative tool for patient stratification. Here, we construct and present a uniquely fine‐grained model of temporal progression of Huntington's disease from premanifest through to manifest stages.

**Methods:**

We employ a probabilistic event‐based model to determine the sequence of appearance of atrophy in brain volumes, learned from structural MRI in the Track‐HD study, as well as to estimate the uncertainty in the ordering. We use longitudinal and phenotypic data to demonstrate the utility of the patient staging system that the resulting model provides.

**Results:**

The model recovers the following order of detectable changes in brain region volumes: putamen, caudate, pallidum, insula white matter, nonventricular cerebrospinal fluid, amygdala, optic chiasm, third ventricle, posterior insula, and basal forebrain. This ordering is mostly preserved even under cross‐validation of the uncertainty in the event sequence. Longitudinal analysis performed using 6 years of follow‐up data from baseline confirms efficacy of the model, as subjects consistently move to later stages with time, and significant correlations are observed between the estimated stages and nonimaging phenotypic markers.

**Interpretation:**

We used a data‐driven method to provide new insight into Huntington's disease progression as well as new power to stage and predict conversion. Our results highlight the potential of disease progression models, such as the event‐based model, to provide new insight into Huntington's disease progression and to support fine‐grained patient stratification for future precision medicine in Huntington's disease.

## Introduction

Huntington's disease (HD) is a monogenic, autosomal‐dominant neurological disorder characterized by motor, cognitive, and behavioral symptoms that have a devastating effect on the life of the person affected.^1^ Symptoms typically begin in early adult life and the disease is usually fatal, with a median survival rate of 18 years after motor onset.^2^ Despite the disease being identifiable by a single genetic marker – an expanded cytosine‐adenine‐guanine (CAG) repeat in the huntingtin gene^3^ – an effective disease‐modifying treatment has yet to be found. This is complicated by the difficulty in assigning gene‐positive subjects to suitable groups when conducting drug trials; within any group there may be a range of physiological and biophysical factors that cause a very different response to treatment. Furthermore, HD displays an extended premanifest period^4^, ^5^ during which therapeutic intervention is likely to be most effective. Identifying a suitable biomarker that captures disease stage may potentially aid trial efficiency when included in a stratified statistical analysis. While there are a range of available biomarkers from different sources – such as functional, imaging, and genetic data – that give some information on disease stage, none is accurate enough alone to be useful for stratification. The challenge therefore is to provide methods that can integrate information from multiple biomarkers for reliable stratification of homogeneous subgroups.

The emerging field of disease progression modeling, in which data‐driven statistical models are designed to both describe long‐term progression of the disease in terms of a set of biomarkers and to categories the stages along that progression of individual patients, provides a potential solution to this challenge; a brief overview is given here, for a more comprehensive review see.^6^ Hypothetical models such as those originally proposed by^7^ describe progression in terms of key biomarker trajectories, typically defined as the monotonic transition from a normal to abnormal state in an order characteristic of the disease, where the order is hypothesized on aggregated evidence from the literature. As such, models of this type represent a qualitative, high‐level view of the disease that do not make direct use of subject data, thus limiting their staging and prediction capabilities in practice.

The event‐based model (EBM;^8^) is a probabilistic method that infers the order in which biomarkers become abnormal directly from cross‐sectional subject data. Its key strengths are (1) it requires no a priori staging, (2) it requires no a priori cut points (i.e., imposed thresholds) to define normal from abnormal, and (3) its simplicity: it can be completely specified using only modestly sized cross‐sectional data (although it can also exploit longitudinal data). Strengths (1) and (2), in particular, set the EBM apart from more traditional region‐based analyses in HD, for example,^9^ which need to assign a stage to each subject a priori, typically using ranges (cut‐points) of an independent marker such as a cognitive test score, to elicit any temporal information on the order in which volumes become detectably abnormal. That limits model resolution and integrity, as the a priori staging is imperfect. The EBM provides a simple and robust tool for investigating disease patterns and estimating patient stages in a fully data‐driven manner. In familial and sporadic Alzheimer's disease,^8^, ^10^ as well as frontotemporal dementia,^11^ EBMs have provided staging systems with predictive power at least as good as pattern matching techniques, for example.^12^, ^13^ In contrast to pattern matching, however, the EBMs provide uniquely fine‐grained temporal patterns of atrophy, enhancing disease understanding, and a well‐defined staging system for stratification. Furthermore, we highlight that the EBM advances on more traditional region‐based analyses,^9^ as it provides temporal information on the order in which volumes become detectably abnormal.

Here, we construct an EBM from the extensive and high‐quality TRACK‐HD dataset (Table 1).^5^, ^14^ This provides a uniquely fine‐grained data‐driven sequence of the regional appearance of brain volume abnormalities in HD and an image‐based staging system. Although the original EBM methods paper^8^ shows a HD model, it is for demonstration purposes only, using a small single‐centre study, and the work makes no evaluation of the potential for staging and prediction. The much larger dataset we use here allows us to specify an EBM with a well‐defined ordering of events and demonstrate that it is robust under cross‐validation. Interestingly, our approach reveals that the spread of atrophy in early‐stage HD is not limited to the basal ganglia and white matter (compared to prodromal observations, e.g., 15), but that changes in these volumes occur first. We show the novel staging system, the resulting EBM provides, gives strong prediction of conversion using only imaging data. We also show that the staging system can separate subjects within the premanifest cohort according to predicted onset, which is independently prescribed by a disease burden score. We confirm the longitudinal efficacy of the model by showing that patients consistently move to later stages with time, using additional data from the TrackOn‐HD study^16^ to provide data 6 years from baseline. We further validate and contextualize the EBM by showing significant correlations between its staging predictions and commonly used phenotypic markers, specifically: total motor score, symbol digit modalities test, and Stroop word reading test. As such, our model provides new insight into the spread of pathology over the brain in HD and new utility in patient stratification for future precision medicine.

**Table 1:** Baseline demographic data for the TRACK‐HD cohort

Demographics	Healthy controls	Premanifest	Manifest
*N*	119	120	118
Gender M/F	53/66	54/66	54/64
Age (years, mean ± SD)	46.3 ± 10.2	40.8 ± 8.8	48.5 ± 9.9
Education (ISCED rating, mean ± SD)	4.0 ± 1.3	3.94 ± 1.2	3.65 ± 1.3
CAG (repeats, mean ± SD)	N/A	43.1 ± 2.4	43.7 ± 3.0
Total intracranial volume (mL, mean ± SD)	1392 ± 136	1408 ± 151	1362 ± 130

The TRACK‐ON cohort used in this study is a subset of 91 of the premanifest subjects at baseline. No significant differences in demographic data were found between the TRACK‐ON subset and TRACK‐HD, except the gender ratio which is approximately 0.7 in the former and 0.8 in the latter.

## Materials and Methods

### Subjects

The TRACK‐HD dataset was used in all following analyses.^5^, ^14^ This study uses a total of 357 subjects with clinical diagnoses at baseline and quality‐controlled imaging data – 119 healthy control (HC), 120 premanifest (pre‐HD), and 118 manifest (HD) – from four different sites: Leiden (NL), London (UK), Paris (FR), and Vancouver (CA) (Table 1). For detailed demographic information see Table 1. Longitudinal data for both the pre‐HD and HD cohorts are available for three follow‐up years in the TRACK‐HD study. Additional data 6 years from baseline are supplied by the TrackOn‐HD study, which contains 69 of the HC and 91 of the pre‐HD subjects present at baseline in TRACK‐HD. The TrackOn‐HD study followed the pre‐HD and control cohorts with the aim of understanding the compensatory mechanisms that allow maintenance of function in the presence of structural loss in the brain. The longitudinal data are reserved for validation purposes as the EBM requires only cross‐sectional data. Both imaging and phenotypic data are available for most subjects; here, we focus on the former to construct the EBM and use the latter for validation and to relate the EBM to clinical measures.

### Magnetic resonance imaging

Postprocessing of multisite 3T T1‐weighted MRI was performed to acquire cross‐sectional regional measurements of brain volumes. The Geodesic Information Flows (GIF) software framework^17^ was used to segment and parcellate cortical and subcortical volumes. The GIF framework provides a more robust segmentation than other state‐of‐the‐art methods such as Freesurfer (http://surfer.nmr.mgh.harvard.edu/), which has been shown to produce noisy segmentations in some regions, for example, the putamen.^18^, ^19^

### Biomarker selection

Herein, we use the term biomarker to refer to image‐based regional brain volumes that differentiate between healthy controls (HC) and HD subjects. To control for covariates, all cortical and subcortical volumes were corrected for age, research site, and total intracranial volume (TIV) using linear regression. To select covariate‐corrected volumes in a data‐driven manner, a group‐wise analysis was performed between the healthy control (HC) and HD cohorts. This approach was partly motivated by aiming to make no a priori decision on the relevance of a given volume, and to provide the EBM with well‐defined normal and abnormal distributions. Significant and strongly separated volumes were defined as having *P*‐value *P *<* *0.001 (corrected for multiple comparisons) and effect size |t| > 8.0, under a two‐tailed *t*‐test. This identified the following brain volumes (here, where relevant, ‘l’ corresponds to the left volume and ‘r’ corresponds to the right volume): putamen (l‐r), caudate (l‐r), amygdala (l‐r), pallidum (l‐r), CSF, insula white matter (l‐r), third ventricle, optic chiasm, posterior insula (l‐r), and basal forebrain (l‐r). Bilateral volumes were only selected if both left and right volumes passed the requirements. This excluded the occipital pole and occipital gyrus, in which the right but not the left volumes passed the effect size requirement. We note that while it is not safe to assume that HD affects the brain symmetrically (see, e.g., 20), these volumes also failed the effect size criterion after their bilateral volumes were combined. Furthermore, the exclusion of any particular volume does not bias the overall biomarker ordering recovered by the EBM; the relative positions of any of the other volumes remain unchanged, and hence it just subtracts knowledge of the position of that particular volume in the sequence. On the other hand, including insensitive biomarkers (i.e., those that do no separate patients and controls) in the model can cause bias and counterintuitive effects. Thus, here we take the conservative approach of excluding any volumes over which the whole volume (left + right) does not sufficiently discriminate between HC and HD subjects.

We note that each of these biomarkers shows a measurable difference in brain volume between HC and HD subject. This does not imply that the change is biologically plausible, for example, it is unlikely that the optic chiasm is directly atrophied by the disease itself. We simply assert that as the disease progresses there is a measurable change in each volume, and that this change is due to disease progression and not any of the aforementioned covariates.

### The event‐based model

The EBM method^8^, ^10^ models a disease process as a sequence of events at which individual biomarkers become abnormal, based on the assumptions of monotonic and homogeneous disease progression, that is, patients experience no remission and all follow the same pattern. It is probabilistic by design, learning normal and abnormal distributions of each biomarker from the data, and requires no a priori staging or cut points. It learns the sequence of events from either or both cross‐sectional and longitudinal datasets and, importantly for clinical trials, enables the assignment of a stage to a subject using data from a single time point.

In brief, the EBM fits a mixture model to control and patient data for each biomarker to obtain models for the distribution of normal and abnormal values for each biomarker. These models provide the likelihoods $P\left(x_{\mathrm{ij}}|E_{i}\right)$and $P\left(x_{\mathrm{ij}}|\neg E_{i}\right)$ of observing the value, $x_{\mathrm{ij}}$, of biomarker *i* for subject *j*, given that biomarker *i* has or has not become abnormal, respectively. These likelihoods combine to calculate the likelihood of the full dataset $X=x_{\mathrm{ij}}:i=1,\ldots ,Z;j=1,\ldots N$ for a given sequence, *S*:

(1) $$P\left(X|S\right)=\prod_{j=1}^{N}\left[\sum_{k=0}^{Z}\left(P\left(k\right)\prod_{i=1}^{k}P\left(x_{\mathrm{ij}}|E_{i}\right)\prod_{i=k+1}^{Z}P\left(x_{\mathrm{ij}}|\neg E_{i}\right)\right)\right]$$

Here, $P\left(k\right)$is the prior likelihood of being at stage *k,* which is assumed uniform to impose as little constraint as possible, *i* runs over the number of events, *Z*, and *j* the number of subjects, *N*. The estimation process then seeks the characteristic sequence, $\overset{¯}{S}$, defined as the sequence that maximizes Equation (1). We use Markov Chain Monte Carlo (MCMC) sampling to sample from the posterior distribution on *S* and identify $\overset{¯}{S}$, which is given by the sample with the highest likelihood. The set of samples further quantifies the uncertainty in the ordering; following,^8^, ^10^ we use the set of MCMC samples to construct a positional variance diagram, which visualizes the uncertainty.

### Models of event distributions

The likelihood models $P\left(x_{\mathrm{ij}}|E_{i}\right)$ and $P\left(x_{\mathrm{ij}}|\neg E_{i}\right)$ were obtained by fitting mixture models to the observed distributions of control and patient regional volumes. We first fit a Gaussian distribution to the set of volumes from the HCs, which provides the model for each $P\left(x_{\mathrm{ij}}|\neg E_{i}\right)$. We then fit a mixture of two Gaussian distributions to the set of volumes from the patients with one component fixed to the HC model's parameters. The parameters of the second, fitted, component provide the model for $P\left(x_{\mathrm{ij}}|E_{i}\right)$. As^10^ notes, estimating the two distributions is harder in sporadic diseases where the control and patient groups are not uniquely defined. However, our simple approach is justified here as the control population is exactly determined by genetic testing; as such, there is approaching zero probability of a control subject developing HD, and the distribution can be treated separately.

### Patient staging

Given a characteristic event sequence, $\overset{¯}{S}$, the EBM provides an intrinsic method for staging by evaluating, for each subject *j* with data $X_{j}$, the stage *k* that maximizes the likelihood^10^:

(2) $$P\left(X_{j}|\overset{¯}{S},k\right)=P\left(k\right)\prod_{i=1}^{k}P\left(x_{\mathrm{ij}}|E_{i}\right)\prod_{i=k+1}^{Z}P\left(x_{\mathrm{ij}}|\neg E_{i}\right)$$

As in Equation (1), $P\left(k\right)$ is the prior likelihood of being at stage *k*, which is assumed uniform (no a priori preference for any particular stage). The stage *k* that maximizes Equation (1) defines the EBM stage of subject *j*. Each stage is a highly idealized combination of normal and abnormal biomarker values, but gives a crude picture of where the patient lies along the characteristic sequence.

### Cross‐validation of event sequence

As described previously, MCMC sampling gives some insight into the uncertainty in the event ordering estimated by the EBM. However, this process tends to underestimate the uncertainty^10^; cross‐validation provides a more liberal measure of the uncertainty. Here, this is performed by refitting the mixture models and re‐estimating the event sequence for 100 bootstrap samples of the data. We construct the final positional variance diagram by averaging over the positional variance diagrams from each iteration.

### Longitudinal consistency

We assessed the predictive capabilities of the EBM under the hypothesis that the stage increases with time, in line with our understanding of HD as a progressive disease. This was tested by estimating pre‐HD and HD subjects’ stages at each follow‐up and comparing to baseline. The TRACK‐HD dataset is comprised of baseline measurements and three follow ups: 12, 24, and 36 months from baseline. A further year of follow‐up data for the pre‐HD cohort were provided by the TrackOn‐HD dataset, extending the follow ups to 72 months. The follow‐up data were processed using the same method as the baseline data to produce the same set of biomarkers at each time point.

### Correlation with phenotypic markers

The TRACK‐HD dataset includes a number of cognitive, functional, and motor measures for each subject at baseline and follow‐up. Here, we use the Total Motor Score (TMS),^21^ Symbol Digit Modalities Test (SDMT),^22^ Stroop test, and scaled CAG‐age‐product (CAP)^23^ as widely used markers of disease‐driven motor, cognitive, and genetic onset, respectively. These markers are used to validate the staging under the hypothesis that a subject with a high EBM stage will have poor motor and cognitive scores. Furthermore, given that TMS, SDMT, Stroop, and CAP are regularly used in clinical diagnosis, correlating these markers with EBM stage provides both an implicit time scale for the event sequence and insight into which events have occurred by the time of motor onset.

## Results

### Region‐based volumetric analysis

To aid in understanding the EBM, Figure S1 shows histograms of the HC and HD volume biomarker distributions and the corresponding mixture model fits. The fits provide the parameters for the normal and abnormal likelihoods, $P\left(x_{\mathrm{ij}}|\neg E_{i}\right)$ and $P\left(x_{\mathrm{ij}}|E_{i}\right)$, respectively. Note that the volumes are covariate corrected, and hence show HC distributions with mean zero

As a complementary and more familiar analysis, we also provide measurements of regional volumetric changes and estimated thresholds of abnormality for each brain volume biomarker (see Table S1). This aids in the interpretation of abnormality for each biomarker, and facilitates comparison with more traditional region‐based studies. Table S1 shows the percentage change in the mean between HC and HD distributions, after correcting for covariates (percentage change = HD residual mean/HC mean). To facilitate comparison with other analyses that estimate explicit thresholds for abnormality, Table S1 also shows the percentage change in each volume with respect to the HC mean at arbitrary thresholds, which we define here as the point at which the volume is equally likely to be normal or abnormal. We highlight that these thresholds are not used by the EBM; they are provided here just to illustrate the separation of the distributions. The volumetric changes and estimated thresholds demonstrate good agreement with the literature, where available.^9^, ^24^, ^25^

### Event sequence

Event orders are presented in the form of a positional variance diagram, which shows the maximum likelihood sequence and its uncertainty. As described earlier, only HC and manifest HD subjects were used to construct the biomarker distribution models, but all subjects were used to produce the final event sequences. To obtain robust fits of the distribution models, we removed all data points more than 5 standard deviations from the within‐group mean; in practice, this excludes a single HC subject where the segmentation overestimated the size of several volumes. Cross‐sectional data from a single time point were used, so the resulting EBM does not consider biomarkers that require follow‐up scans (e.g., atrophy rates). This ensures that data from a single time point can be used to stage subjects.

**Figure 1:**
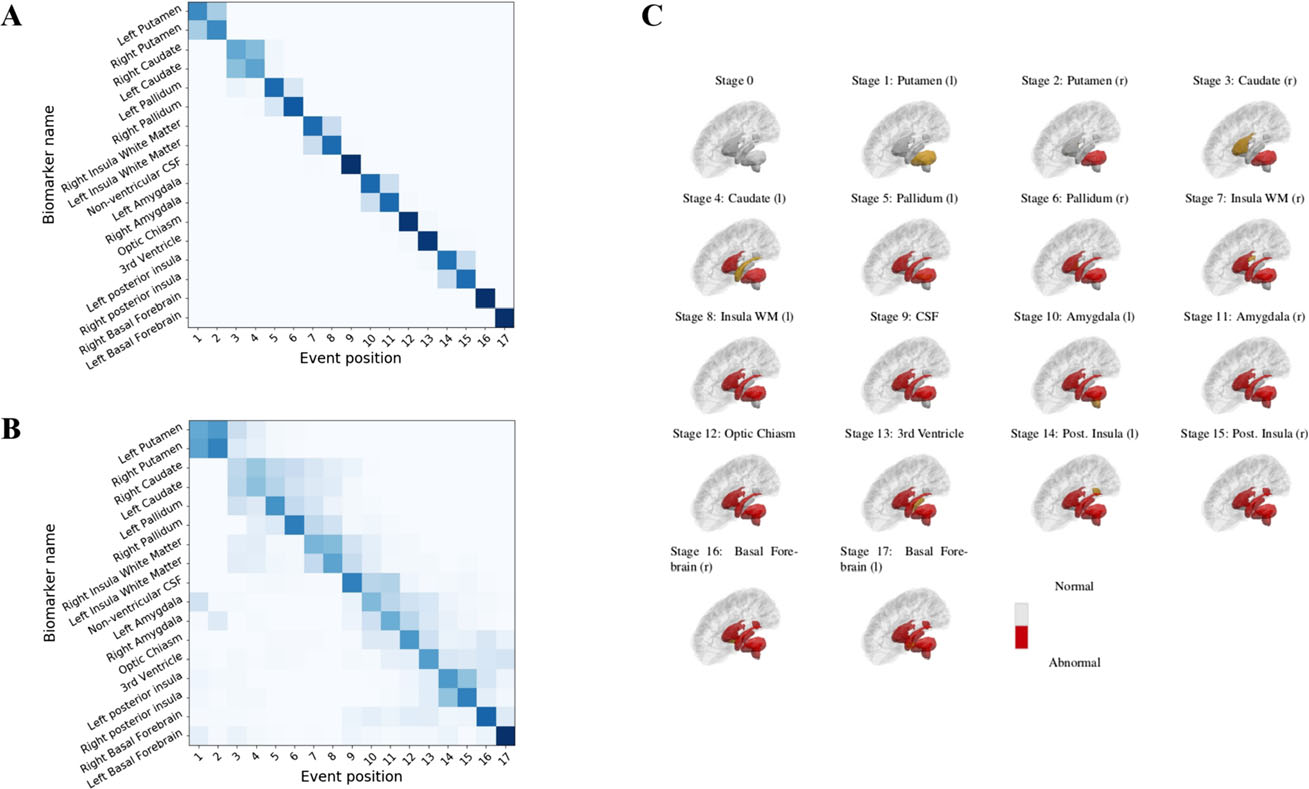
(A) Regional volume biomarker positional variance diagram. Dark diagonal components indicate strong event ordering, and lighter indicate possible event permutations with strength proportional to the off‐diagonal components. (B) Re‐estimation of the positional variance for 100 bootstrap samples of the data. (C) Graphic representation of the event sequence showing the corresponding subcortical regions transitioning from an initially healthy (grey) state to an unhealthy (red) state. To aid in visualization, the newly added region at each stage is colored in orange.

The brain volume biomarker positional variance diagram is shown in (Fig. 1A). Specifically, the order of changes in volume is as follows: putamen, caudate, pallidum, insula white matter, nonventricular CSF, amygdala, optic chiasm, third ventricle, followed by the posterior insula, and finally the basal forebrain. The corresponding positional variance estimated by bootstrapping is shown in (Fig. 1B). To aid in visualization, (Fig. 1C) shows a graphical representation of the event sequence.

### Cross‐validation

Figure 1B shows the positional variance of the maximum likelihood event sequence recalculated by bootstrapping the data (i.e., random sampling with replacement) and refitting the mixture models and sequence. This provides an estimation of the effect of out‐of‐sample variability and hence an overestimation of the uncertainty. The ordering is generally well preserved, with the components of the basal ganglia occurring first, the white matter and CSF retaining a mid‐stage position, and the posterior insula and basal forebrain occurring last.

### Staging

**Figure 2:**
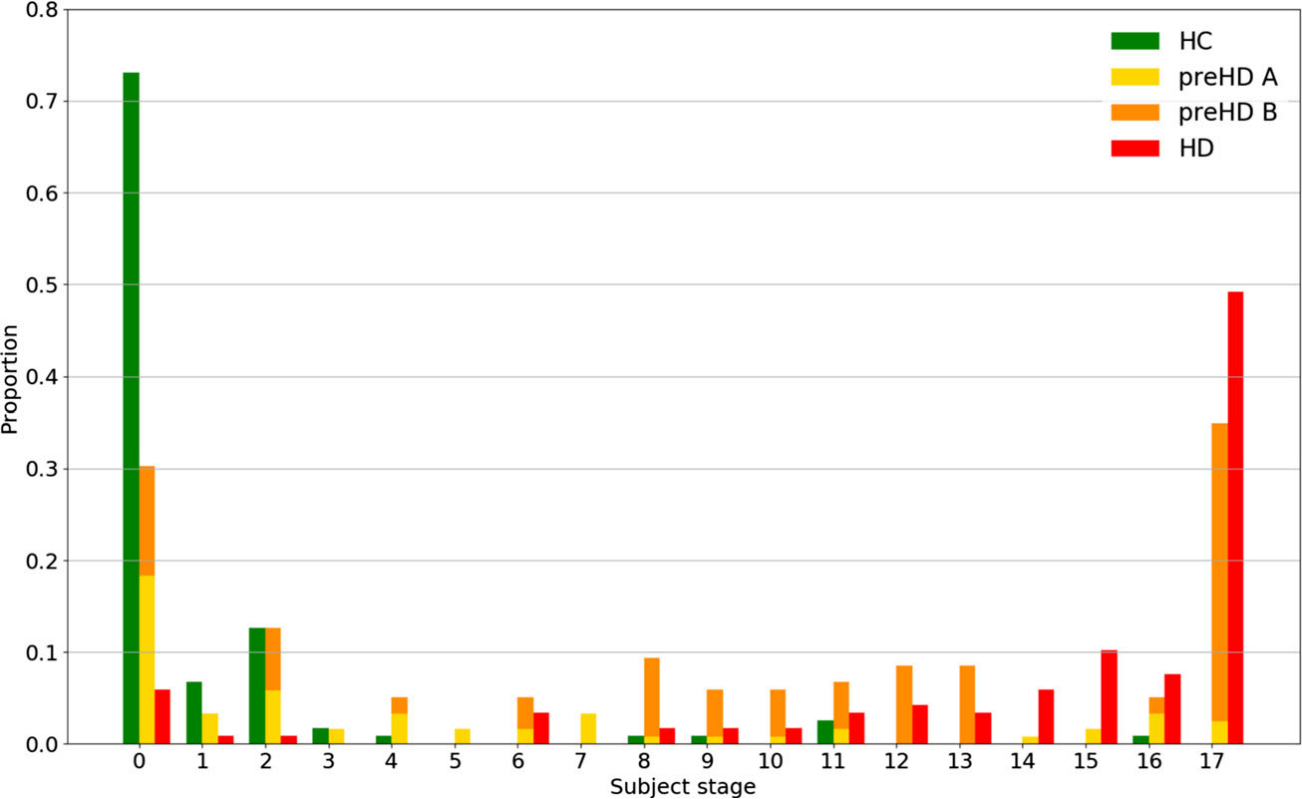
Distribution of subject stages: healthy controls (HC), premanifest A (pre‐HD A), premanifest B (pre‐HD B), and manifest (HD). The proportion is with respect to the total of each group: HC, pre‐HD A + pre‐HD B, and HD.

Subjects were assigned a stage along the maximum likelihood sequence defined in (Fig. 1A) according to (Eq. (1)). The fraction of subjects at each event stage for each cohort is shown in (Fig. 2). The HC cohort is clustered at the early stages, with the majority at stage 0 (no event occurred), whereas the HD cohort is clustered at the late stages, with the majority at the final stage (all events occurred). This demonstrates that the EBM can provide separation between healthy and manifest subjects. The pre‐HD cohort is distributed throughout the sequence with peaks at the first and final events, indicating that premanifest subjects can be grouped as more “HC‐like” or more “HD‐like”. We further substantiate this by dividing the pre‐HD cohort into two subgroups, pre‐HD A and pre‐HD B, which are defined by the TRACK‐HD study according to the predicted time to onset.^5^ The predicted time to onset is based on an age‐ and CAG‐dependent empirical relation,^26^ with pre‐HD A subjects having predicted onset greater than 10.8 years and pre‐HD B subjects less than 10.8 years. Accordingly, the EBM assigns most (77%) pre‐HD A subjects to the lower half of the stages and most (67%) pre‐HD B subjects to the upper half of the stages.

Outliers appear in both the HC and HD groups, specifically one HC subject at stage 18 and the HD subjects at stages 0, 1, and 2. Closer inspection of these subjects suggests their MRIs and regional volumes generally agree better with those of opposite diagnosis, rather than, for example, a single anomalous regional volume or image processing error. The HC at stage 18 showed higher likelihoods of being abnormal than normal with most of their regional volumes agreeing better with $P\left(x_{\mathrm{ij}}|E_{i}\right)$ than $P\left(x_{\mathrm{ij}}|\neg E_{i}\right)$. We note that the HC at stage 18 is 64.9 years old, almost 2 standard deviations greater than the mean HC age; this may explain the anomaly even after correcting for age (which just represents a best fit across all subjects). The HD outliers are assigned a stage less than four because their putamen, caudate, and pallidum all have volumes in the normal range ($P\left(x_{\mathrm{ij}}|\neg E_{i}\right)$>$P\left(x_{\mathrm{ij}}|E_{i}\right)$). Visual inspection confirms no significant parcellation errors, suggesting that the positions of these subjects arise simply from the broad heterogeneity of brain structure and disease manifestation.

### Longitudinal consistency

**Figure 3:**
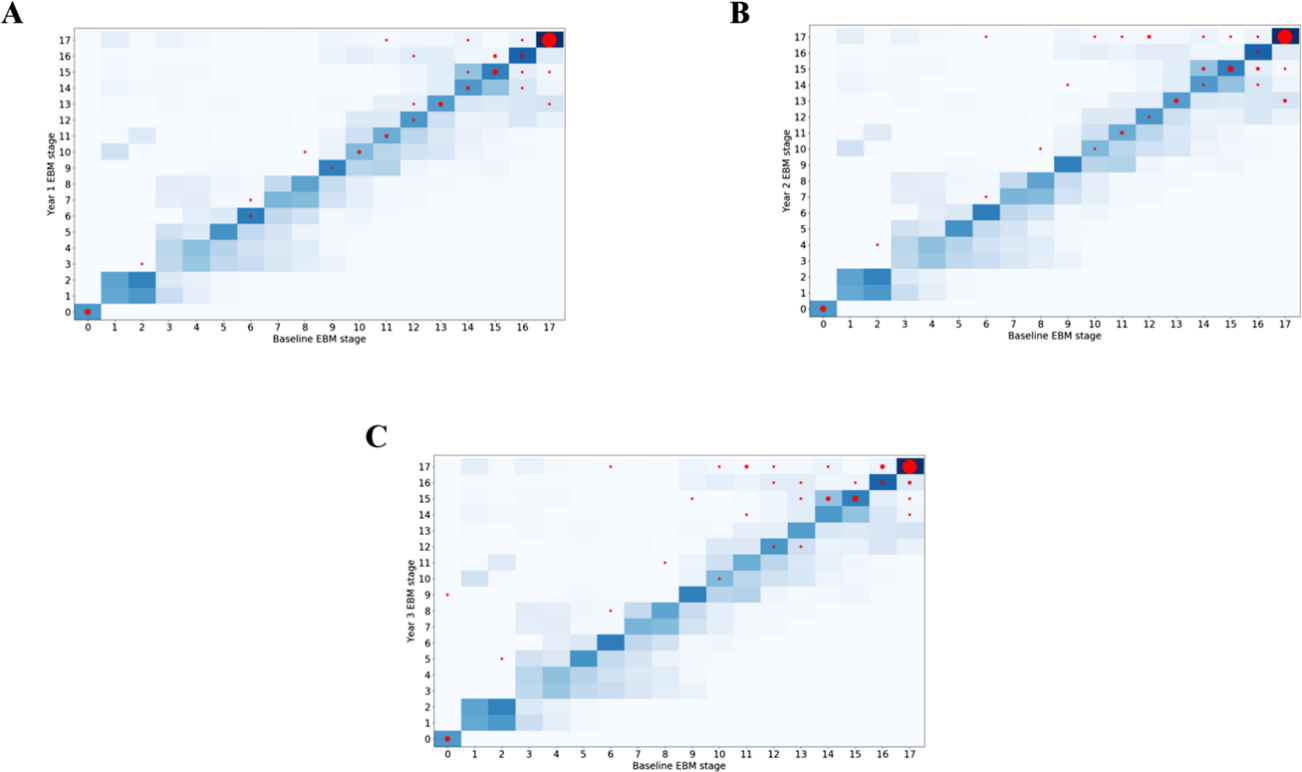
Predicted stage at baseline versus predicted stage at 1 year (A), 2 years (B), and 3 years (C) for the manifest cohort in TRACK‐HD. Predicted stages are shown as red circles (area scaled by the number of entries at each point). The uncertainty in the event ordering – equal to that of the bootstrapped EBM positional variance – is shown as a two‐dimensional heatmap.

The longitudinal consistency of the EBM staging system was first tested using follow‐up data from three consecutive years. (Fig. 3A–C) show the EBM stages at baseline versus the EBM stages at each follow‐up (year 1, 2, and 3) along with the bootstrapped positional variance in the event sequence from (Fig. 1B). It confirms that the subject stages generally increase, stay constant with time, or lie within model uncertainty, as expected. At the final year only three subjects regress more than a single stage from baseline; all of these were at the final stage at baseline. Closer inspection reveals that the small fluctuations in stage arise from the fluctuation of estimated basal forebrain volumes at either baseline or follow‐up. These volumes are prone to causing model error as the HC distribution's standard deviation is larger than that of the HD distribution (approximately a factor of 1.3). The additional follow‐up data provided by the TrackOn‐HD study were included to provide a further 3 years of validation for most (91/120) of the premanifest cohort. A generally increasing trend of stage with time was observed, with 92% of patients staged greater than or equal to their baseline stage at the final follow‐up. Rounding up, the average progression was three stages over 3 years in TRACK‐HD (*n* = 73) and six stages over 6 years in TrackOn‐HD (*n* = 35); average regression was two stages over 3 years in TRACK‐HD (*n* = 17) and two stages over 6 years in TrackOn‐HD (*n* = 6). This indicates that patients progress at approximately one stage per year, and those who regress in stage do so by a maximum of two stages, which is within the uncertainty in the staging mechanism.

### Prediction of conversion

The EBM can also be used to predict conversion from pre‐HD to HD status. We define true converters as patients with a premanifest diagnosis at baseline and an HD diagnosis at year 3 (no conversions were observed at years 1 or 2), and predicted converters as patients with a stage greater than a threshold EBM stage. The balanced accuracy, which is the average of sensitivity and specificity, is then maximized over all stage thresholds. This gives a maximum balanced accuracy of 65% (75% sensitivity; 55% specificity) for predicting converters with a baseline stage greater than 7. While this might not appear particularly high, it is worth noting that only cross‐sectional imaging data were used to power this prediction. By way of comparison, support vector machines (SVM) with linear and nonlinear kernels were trained and tested on the same pre‐HD data. Due to the small number of converters in the sample the SVM could not improve beyond the baseline model of just predicting the same class at follow‐up as at baseline. This highlights the ability of the EBM to draw useful predictive information using only cross‐sectional data. Furthermore, the predictive power is likely to be increased by including longitudinal information in the EBM, such as the rate of atrophy.^10^

### Correlation with phenotypic markers

**Figure 4:**
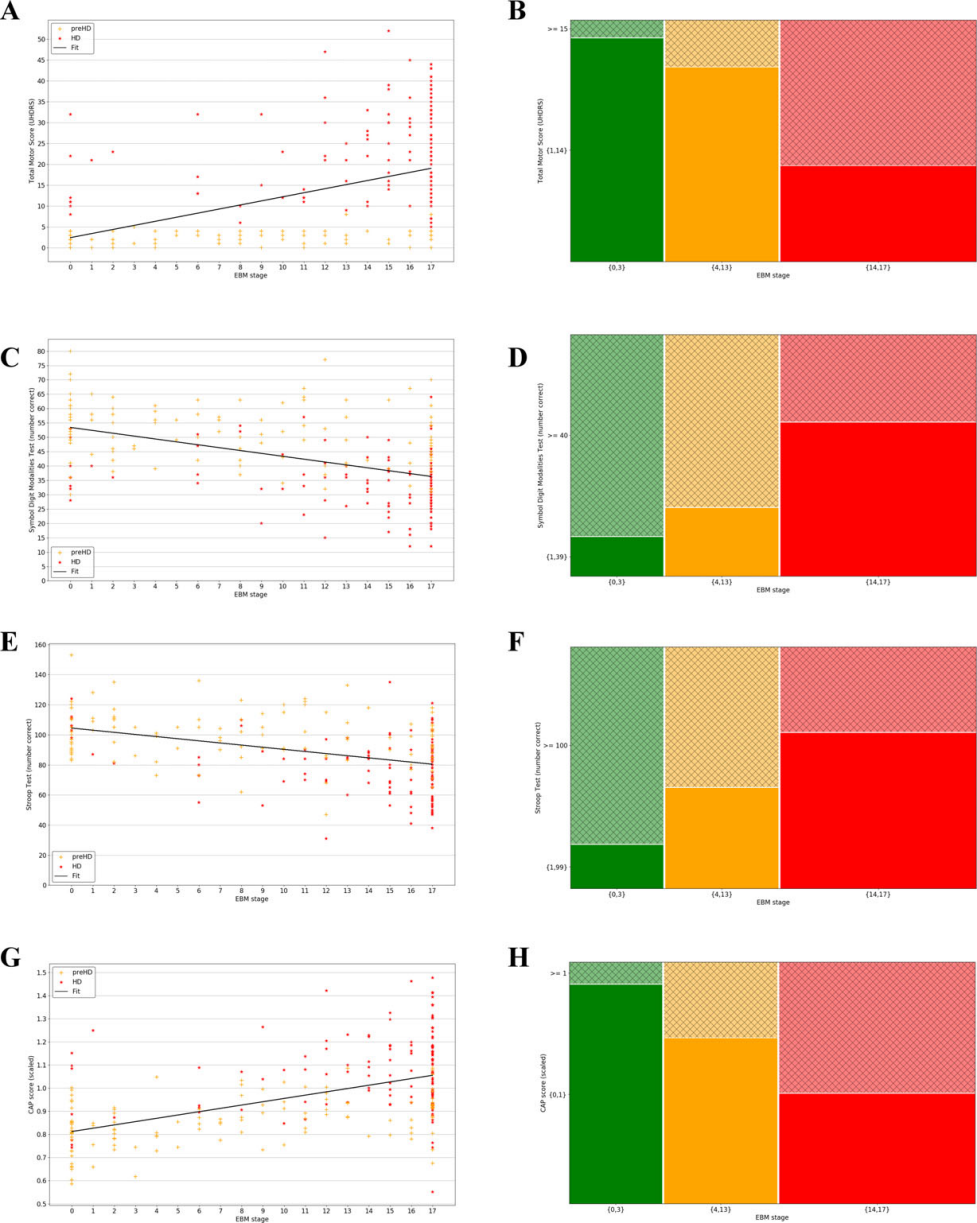
(A) Total motor score (TMS) versus event‐based model (EBM) stage plus linear model fit to both pre‐HD and HD subjects; (B) Symbol Digit Modalities Test (SDMT) versus EBM stage plus linear model fit to both pre‐HD and HD subjects; (C) Stroop word reading test versus EBM stage plus linear model fit to both pre‐HD and HD subjects; (D) scaled CAP score versus EBM stage plus linear model fit to both pre‐HD and HD subjects; (E) TMS versus EBM stage brackets; (F) SDMT versus EBM stage brackets; (G) Stroop versus EBM stage brackets; (H) scaled CAP score versus EBM stage brackets. All plots show data from the premanifest (pre‐HD) and manifest (HD) groups. The mosaic plots (E–H) show the lower *y*‐axis bracket in solid color and the higher *y*‐axis bracket in thatch, and the number of subjects in each bracket is proportional to its area.

To further validate the EBM, the predicted stages for pre‐HD and HD subjects were plotted as a function of three widely used clinical markers: the total motor score (TMS), symbol digit modalities test (SDMT), and Stroop test. (Fig. 4A) shows a scatter plot of TMS versus stage, (Fig. 4B) the equivalent for SDMT, (Fig. 4C) the equivalent for Stroop, and (Fig. 4D) the equivalent for CAP, for each cohort separately. Ordinary least squares linear regression was performed on the combined cohort in each case, which showed significant gradients (*P *<* *0.001) in the expected directions: TMS (*β *= 0.98) and CAP (*β *= 0.014) increase with increasing EBM stage, SDMT (*β *= −1.0) and Stroop (*β *= −1.4) decrease with increasing EBM stage. This demonstrates the potential clinical relevance of EBM stages despite being estimated purely from volumetric MRI data.

In addition to a means of validation, the linear models in (Fig. 4A–D) allow the EBM staging to be related to potential thresholds for motor and cognitive onset. Example thresholds are chosen to reflect values that separate pre‐HD and HD subjects according to the longitudinal data published by^2^: Figure 2. For thresholds of TMS > 15, SDMT < 40, and Stroop < 100, the EBM predicts motor and cognitive onset for subjects with stage >13. This suggests that by the time of onset, the archetypal subject already has abnormal putamen, caudate, pallidum, insula white matter, CSF, amygdala, optic chiasm, and (with higher uncertainty) third ventricle volumes. According to this model, the posterior insula and basal forebrain transition to an abnormal state after motor onset. It is interesting to note that TMS demonstrates a threshold effect and is only sensitive to changes in HD subjects, not pre‐HD, that is, the pre‐HD data are approximately flat, while SDMT, Stroop, and CAP track changes across both cohorts. Mosaic plots representing these data are shown in (Fig. 4E–H) for each phenotype, and show a clear split in the population above or below each threshold. This further substantiates the EBM as a potential method to stratify subjects.

## Discussion

We have presented a uniquely fine‐grained model of temporal progression of volume loss in premanifest and manifest HD that is robust under cross‐validation. The model provides a novel method for prediction of conversion using imaging data alone, and we have demonstrated that the staging can stratify premanifest subjects according to their predicted time to onset. We evidenced the utility of the model via longitudinal validation, and showed that patients consistently move to later stages with time. Finally, we showed that the model staging is significantly correlated with independent phenotypic markers, further supporting the model as a potential tool to support fine‐grained stratification in HD. In the following sections we discuss how the results compare to findings in the literature, where available.

### Ordering of biomarkers

Our model places the putamen, caudate, and pallidum before insula white matter (Fig. 1A), and overall predicts a central‐to‐peripheral pattern of the subcortical spread (see (Fig. 1C) for a visual representation). This is in agreement with recent work by^27^ who used change point linear regression based on a priori disease staging to estimate the initial time of atrophy of structural MRI in the PREDICT‐HD dataset, and observed a central‐to‐peripheral pattern of atrophy from the basal ganglia to deep white matter. The model we present here is drawn from a broader spectrum of stages in the patient cohort than in,^27^ facilitating a more complete picture of the whole disease time course, and avoids the confounds of a priori staging.

Our findings agree well with previous studies, where available. The early involvement of the striatum is well reported.^28^, ^29^, ^30^ Early changes in the basal ganglia nuclei – here represented by the putamen, caudate, and pallidum – are in agreement with previous observations in both premanifest and manifest HD subjects using the TRACK‐HD dataset,^24^ and with other studies based on the putamen and caudate.^29^, ^30^ Their ordering, however, has not previously been observed: the EBM places the putamen strongly ahead of the caudate and pallidum, even under bootstrapping. Abnormalities in the insula white matter and nonventricular CSF are identified by the EBM as potential mid‐stage biomarkers, the former of which is also reported in the PREDICT‐HD dataset,^27^ and agrees with observations of white matter abnormality in premanifest and manifest subjects.^31^, ^32^, ^33^, ^34^

To our knowledge, no data exist on the relative positioning of the remaining brain volume biomarkers. CSF has recently been associated with seeding aggregation of mutant Huntingtin,^35^ and various components have been posited for use as clinical biomarkers.^36^ The EBM then predicts changes in the amygdala, optic chiasm, and third ventricle. The amygdala is topologically connected to the caudate, but the literature concerning its involvement is sparse; where it exists, it focuses on functional tests.^37^ The optic chiasm is not expected to be pathologically involved in the disease progression, but may undergo changes in volume due to the atrophy of surrounding tissues. Measurements of the volume of the third ventricle by transcranial sonography have shown that it is significantly larger in HD patients than HCs.^38^ Finally, the EBM predicts the posterior insula and basal forebrain as late‐stage biomarkers. The posterior insula has been shown to be functionally connected to the striatum and motor cortex, both of which were identified as displaying measurable atrophy in early manifest HD.^39^ Involvement of the basal forebrain in HD is not well reported in the literature, although its neuronal connectivity to the cerebral cortex and amygdala has been studied in relation to the cholinergic system.^40^

### Ordering uncertainty

The uncertainty in the event ordering, given by the positional variance (Fig. 1A), is most effectively examined using the bootstrapped positional variance diagram shown in (Fig. 1B). These results demonstrate that even with an overestimation of the uncertainty, the event ordering is largely preserved. This is particularly noteworthy given that the model is sensitive to a number of factors present in the data, namely, disease heterogeneity, sampling density, and statistical outliers.^10^

The uncertainty of the relative positions of the caudate and pallidum is high and their positions can effectively be permuted. There are two possible explanations for this: (1) that the events occur simultaneously or interchangeably across subjects; and (2) that our cohort does not include subjects at stages that specify the ordering of these regions. With respect to the pallidum, measurement error may also be an issue, due to its small size.

### Staging system

The EBM provides an intrinsic staging mechanism and hence the means to correlate the model with clinical metrics. The uncertainty the assigned stage depends on (1) the accuracy of a given subject's biomarker measurement, and (2) the degree of overlap between the healthy and manifest distributions. Here, we have partially addressed these factors by statistical testing: the former by removing outliers using, for example, the 5‐sigma cut noted earlier; and the latter by requiring that the *t*‐tests on the HC and HD distributions have a large effect size. We are only using imaging data here and hence expect high heterogeneity leading to outliers; as discussed earlier this is observed in a small number of subjects. Despite this we get strongly predictive staging performance from the EBM: it successfully divides the pre‐HD group into CAG‐ and age‐dependent subgroups defined by the TRACK‐HD study, can perform predictions of conversion using only cross‐sectional imaging data, and has good longitudinal consistency over 6 years. There is still a degree of uncertainty associated with each subject's stage, however; this is reflected in the longitudinal validation, which shows a small number of subjects regressing from their baseline stage.

The EBM presented here uses only image‐based data, thus allowing a noncircular relationship between imaging and phenotypic markers to be established. The results shown in (Fig. 4) suggest an intersection of quantitative image‐based stratification and traditional clinical‐based metrics. While the thresholds used here were chosen to split pre‐HD and HD subjects into separate groups, any number of subgroups can be defined according to the desired level of stratification. Given the variability in the pre‐HD subject stages (Fig. 2), this type of correlation can provide insight into potential heterogeneity in clinical trial groups.

### Limitations and future work

As noted in^10^, the EBM makes model assumptions that must be considered when interpreting these results. The strong assumption that the disease progression is homogeneous across the population is common in disease progression modelling.^6^ While this assumption may not be as much of a simplification in HD (which is caused by a single genetic mutation) as it is in Alzheimer's disease (which typically exhibits much greater heterogeneity), it is reasonable to assume that the complex cascade of both damaging and compensatory events associated with HD causes different pathologies. Despite the assumption of a single pattern, which is likely to be overly simplistic, the EBM demonstrates useful staging capability, suggesting that at least some aspects of the progression pattern are broadly shared by most patients. Newer techniques such as subtype and stage inference^42^ potentially reveal distinct patterns and may provide even greater predictive ability in the future. In addition, as discussed by,^42^ the EBM assumes independence of biomarkers, which can affect the estimation of biomarker event distributions. Here, we have a well‐defined HC population and strongly separated means between the HD and HC populations, allowing for good estimation of the biomarker event distributions, so this effect should be negligible.

The EBM has no time dependence and hence no explicit time scale; it can therefore predict where a given subject is in the disease sequence, but not how long it has taken to reach that stage. The advantage of this is that the EBM requires only cross‐sectional data, which enhances its clinical utility. Here, we gave the EBM an implicit time scale in a qualitative manner by correlating the predicted stages with phenotypic markers; the time course of a given subject's total motor score, for example, can be measured and related back to the staging. This is only an approximation, and there are associated errors with both the stage and phenotype.

To lend the disease progression an explicit time scale a different model could be applied that utilizes longitudinal data (see 44 for a more comprehensive review). Trajectory‐based models that are informed by patient data have been proposed that employ linear mixed models^44^ or quantile regression,^45^ with the latter allowing for nonmonotonic trajectories. However, these models require an a priori definition of disease stage; in the cited examples, this is expected age to onset and time to diagnosis, respectively, both of which need to be estimated. In practice, these estimates are crude, for example, from parental age of onset, which can lead to inaccuracy of the model. Trajectory modeling without the need for a predefined disease stage can be achieved using differential equation models^46^, ^47^ and self‐modeling regression techniques^48^ or Gaussian Processes.^43^ Such models provide complete temporal pictures of disease progression at the expense of requiring data from at least two time points to parameterize.

Future work on validating the model across multiple datasets will be valuable for translation to clinical practice. We plan to use data from the PREDICT‐HD study to test the model, as it represents a different cohort and will allow us to probe the effects of population variance. Furthermore, we will specify models to each study separately and compare the predicted progression patterns.

## Conclusions

We have presented a data‐driven model of brain volume biomarker changes in Huntington's disease that shows good longitudinal consistency and potential clinical applicability. The model proposes a characteristic sequence of events with a strong ordering that provides insight into Huntington's disease progression and a potential tool for patient stratification.

## Author contributions

PAW, ALY, NPO, and DCA conceived and designed the study. All authors contributed to acquiring or analyzing the data. PAW drafted the manuscript and figures.

## Conflicts of Interest

The authors declare that there are no conflicts of interest present in this work.

## Supporting information


**Figure S1.** HC (green) and HD (red) volume biomarker distributions, and corresponding mixture model fits. Note that the volumes are covariate corrected.

**Table S1.** Percentage change in mean volumes and thresholds between healthy control (HC) and manifest (HD) regional brain volume distributions, after controlling for covariates (age, site, and total intracranial volume).
