# Supplementary material for: An image‐based model of brain volume biomarker changes in Huntington's disease
Source: Ann Clin Transl Neurol. 2018 Apr 2;5(5):570–82. doi: 10.1002/acn3.558 (PMC5945962; doi:10.1002/acn3.558)
Supplement: Supplementary file 1 — Figure S1. HC (green) and HD (red) volume biomarker distributions, and corresponding mixture model fits. Note that the volumes are covariate corrected. [file ACN3-5-570-s001.docx]

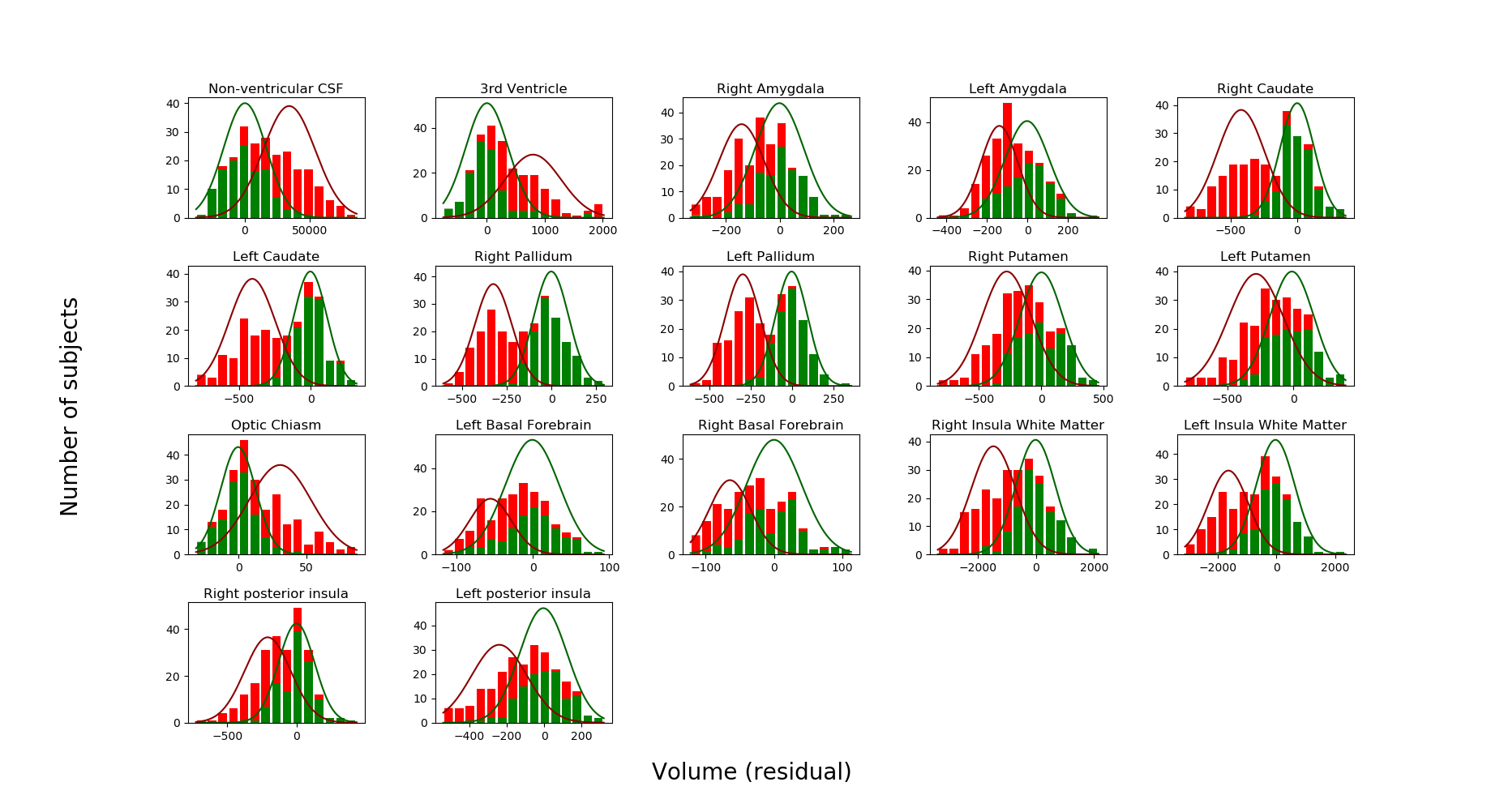
Fig. S1: HC (green) and HD (red) volume biomarker distributions, and corresponding mixture model fits. Note that the volumes are covariate-corrected.
