# Supplementary material for: An image‐based model of brain volume biomarker changes in Huntington's disease
Source: Ann Clin Transl Neurol. 2018 Apr 2;5(5):570–82. doi: 10.1002/acn3.558 (PMC5945962; doi:10.1002/acn3.558)
Supplement: Supplementary file 2 — Table S1. Percentage change in mean volumes and thresholds between healthy control (HC) and manifest (HD) regional brain volume distributions, after controlling for covariates (age, site, and total intracranial volume). [file ACN3-5-570-s002.docx]

| **Regional brain volume** | **Percentage change at threshold (%**$\pm$**SEM)** | **Percentage change in mean (%**$\pm$**SEM)** |
| --- | --- | --- |
| Non-ventricular CSF | 6.5$\pm$1.1 | 14$\pm$5.5 |
| 3rd ventricle | 31$\pm$3.4 | 56$\pm$7.8 |
| Right amygdala | -5.8$\pm$0.9 | -10$\pm$5.9 |
| Left amygdala | -5.2$\pm$0.9 | -9.1$\pm$6.6 |
| Right caudate | -11$\pm$1.1 | -22$\pm$4.4 |
| Left caudate | -11$\pm$1.1 | -22 $\pm$4.3 |
| Right pallidum | -10$\pm$0.9 | -19 $\pm$3.3 |
| Left pallidum | -9.6$\pm$0.9 | -19 $\pm$3.5 |
| Right putamen | -6.2$\pm$1.0 | -10 $\pm$6.6 |
| Left putamen | -6.7$\pm$1.1 | -10 $\pm$7 |
| Optic chiasm | 15$\pm$1.5 | 29$\pm$7.6 |
| Right basal forebrain | -5.9$\pm$0.9 | -8.8$\pm$6.9 |
| Left basal forebrain | -5.9$\pm$0.9 | -7.5$\pm$8.4 |
| Right insula white matter | -5.7$\pm$0.9 | -10$\pm$5 |
| Left insula white matter | -6.3$\pm$0.9 | -10$\pm$5 |
| Right posterior insula | -6.8$\pm$1.0 | -10$\pm$7.8 |
| Left posterior insula | -7.3$\pm$1.1 | -10$\pm$7.8 |

Table S1: Percentage change (PC) in mean volumes and thresholds between healthy control (HC) and manifest (HD) regional brain volume distributions, after controlling for covariates (age, site and total intracranial volume). The percentage change at threshold is defined as the percentage change in volume at which the volume is equally likely to be normal or abnormal, according to our model.
